# Similarities and differences between natural sleep and urethane anesthesia

**DOI:** 10.1038/s41598-025-01762-0

**Published:** 2025-05-25

**Authors:** Jurij Brankačk, Yevgenij Yanovsky, Adriano B. L. Tort, Andreas Draguhn

**Affiliations:** 1 Institute for Physiology and Pathophysiology, Heidelberg University, 69120 Heidelberg, Germany; 2 Brain Institute, Federal University of Rio Grande do Norte, Natal, RN 59078-900 Brazil

**Keywords:** Sleep, Urethane anesthesia, Slow oscillation, δ waves, Parietal cortex, Circadian rhythms and sleep, Learning and memory

## Abstract

Slow oscillations dominate the EEG or local field potential (LFP) of mammals during specific periods within natural sleep and anesthesia. Such similarities have led to the use of anesthesia as a model to study sleep and state-dependent changes of consciousness. Previous research has documented the similarities between the activated state of urethane anesthesia and natural REM sleep, particularly with respect to network oscillations in the theta (θ) frequency domain. Likewise, the deactivated states, characterized by large amplitude slow waves in both urethane anesthesia and non-REM sleep, have generally been regarded as similar. Here, we report striking differences between slow oscillations in the mouse parietal cortex during the deactivated state of urethane anesthesia and natural non-REM sleep. These differences are notable in the LFP, the underlying current sources, and in the modulation of unit activity. Our data show that slow network oscillations in natural sleep and anesthesia are generated by different mechanisms, despite phenomenological similarities.

## Introduction

During the last decade, studies on consciousness^1^ have sought to identify shared brain circuits across natural sleep, anesthesia, coma, and sedation^2–6^. Although all general anesthetics lead to loss of consciousness^7^, they act on different receptors^8^, indicating that their common effects are generated by heterogeneous mechanisms. Similarly, comparisons between anesthesia and sleep have produced diverse and partially contradictory conclusions about the similarities of the respective states^9–16^.

One commonly used approach to explore different states of vigilance is the analysis of EEG recordings in humans or intracerebral local field potentials (LFP) in animals. Electrophysiological signals of the brain are often oscillatory^17^, with frequencies ranging from near 0–600 Hz^18^. The amplitude of each frequency component, like many natural processes, follows a power law (1/f), leading to easily observable large slow waves and less easily discernible low-amplitude high-frequency oscillations^19,20^. Characteristic EEG signatures are the key parameters for identification of brain states like sleep, wakefulness, and anesthesia. These brain “states” are non-stationary; they evolve dynamically and consist of more or less stationary states, such as non-rapid eye movement (NREM) and rapid eye movement (REM) sleep, or quiet and active waking.

Urethane anesthesia is commonly considered a model for sleep^10,13,16^ (but see reference 15). Animals anesthetized with urethane exhibit two main brain states: a deactivated state (dU) characterized by slow waves resembling natural NREM sleep^10,21^, alternating with an activated state (aU) with hippocampal θ activity^22,23^ similar to that seen in REM sleep^10,24^. Deactivated and activated states also appear (depending on the dose) under ketamine-xylazine and chloral hydrate anesthesia, but do not occur under pentobarbital, isoflurane, or propofol^25^.

In the present work, we sought to address whether urethane anesthesia (see Methods for doses and effect duration) provides an accurate model for natural sleep. While previous work showed that the activated state of urethane anesthesia mirrors REM sleep, the comparability of the deactivated urethane state to NREM sleep is less clearly documented, despite a general notion of similarity^10,21^. The main question for this study was whether slow oscillations (SO, 0.1–1.5 Hz) first described in anesthetized cats^21^ can also be found in natural sleep in mice. We performed LFP recordings from the posterior parietal cortex of mice to investigate key similarities and differences between slow waves in the deactivated state of urethane anesthesia and those in natural NREM sleep. By examining LFP patterns and modulation of unit activity, we reveal major differences that limit the validity of urethane anesthesia as a model for NREM sleep.

## Results

Natural sleep is characterized by periods of slow wave activity (1 to 4 Hz), also known as non-rapid-eye-movement (NREM) sleep, alternating with periods lacking slow waves but exhibiting regular θ (4.5–12 Hz) oscillations, known as REM sleep. Interestingly, urethane anesthesia exhibits a similar alternation, with spontaneous periods of slow waves, termed the deactivated state (dU)^10^, alternating with periods of θ oscillations, albeit at a lower frequency (3.5–5 Hz), known as the activated state (aU)^10^. Our results rely on electrophysiological measurements in urethane anesthesia, two hours after the initial application of a low dose of ketamine-xylazine (see Methods for details). The posterior parietal cortex (PAC) was chosen due to the presence of marked θ rhythms in REM sleep, which are volume conducted from the dorsal hippocampus^26^ and lacking in frontal cortical areas. This facilitates sleep–wake staging which depends on the presence of θ rhythm and on motion detection by accelerometry. We first tested whether there is natural sleep during the circadian dark phase (see Methods). In a group of nine male mice, cable recordings revealed natural sleep patterns in 13% of the recorded dark time, with NREM corresponding to 10.4 ± 2.7% and REM to 2.6 ± 1.0%. For a mean recording time in the dark phase of 28836 s, NREM was present for an average of 2761 ± 713 s and REM for 756 ± 366 s. The occurrence of REM sleep after NREM sleep is an indicator that even in the dark phase there is deep NREM sleep.

### Similarities and differences in slow network activity during natural sleep and under urethane anesthesia

**Fig. 1:**
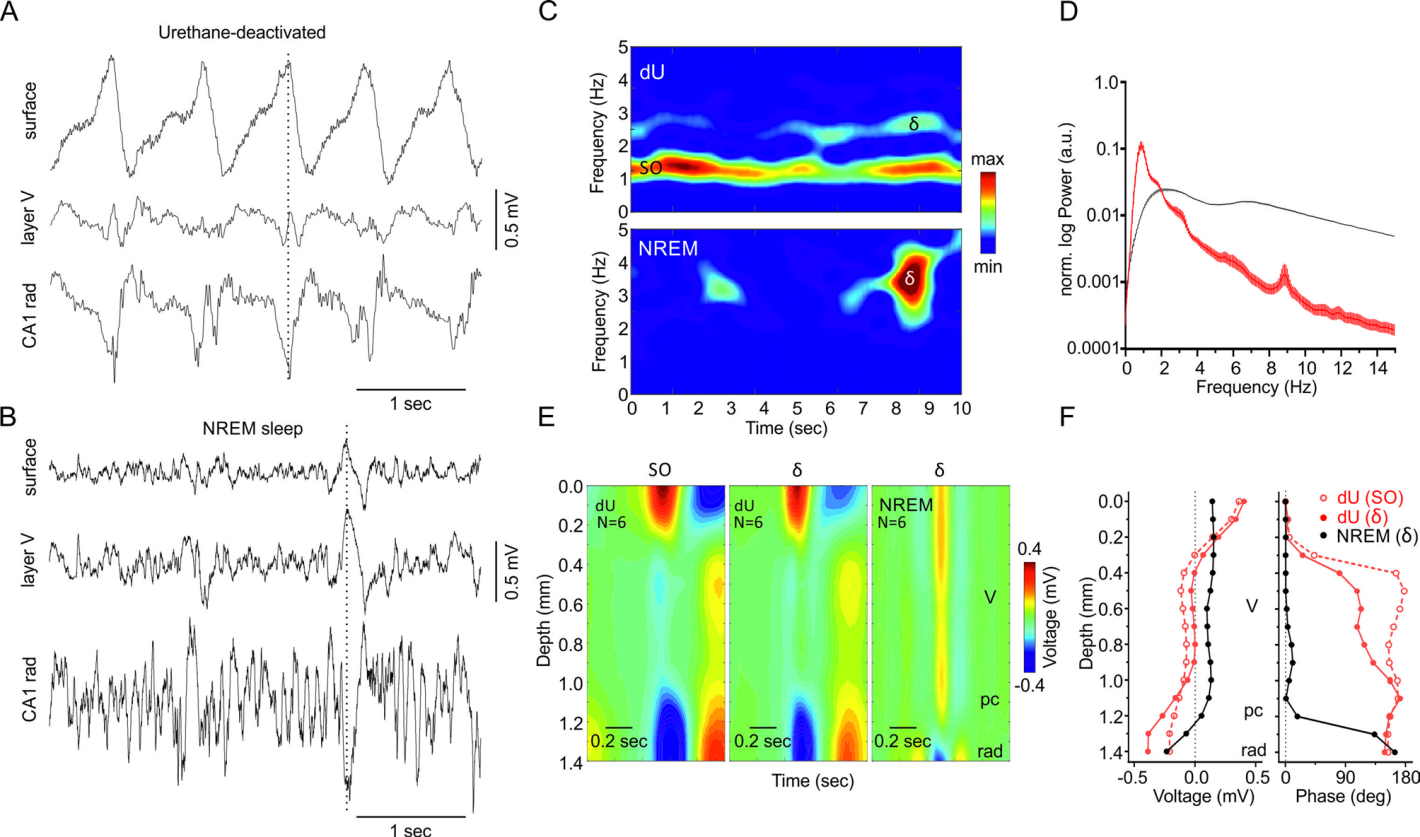
Similarities and differences in slow network activity during natural sleep and under urethane anesthesia. (**A**) Local field potentials (LFPs) recorded in the deactivated state (dU) of urethane anesthesia at the surface (surf, upper trace), layer V (middle trace) of parietal cortex (PAC), and stratum radiatum (CA1 rad) of the dorsal hippocampus. (**B**) LFPs in NREM sleep recorded at the same depths as in A. (**C**) Time–frequency distribution (TFD) of 10 s periods of surface LFP during dU (upper graph) and NREM sleep (lower graph). (**D**) Normalized (norm., see Methods) power spectrum of surface PAC LFP in dU (red) and NREM sleep (black), averaged across 12 animals (mean ± standard errors) displayed in logarithmic scaling. Power in the lower δ range is similar in dU compared to NREM as shown in E. (**E**) Average voltage depth profiles (N = 6 animals) of slow oscillation (SO: 0.1–1.5 Hz, left graph) and δ (1.5–4 Hz) activity (middle graph) in dU, and δ activity in NREM sleep (right graph). (**F**) Mean changes in voltage (left and phase (right, N = 6 animals) with depth during dU (red) and NREM (black). Note the similarities between SO and δ activity in dU and the difference from δ activity in NREM sleep. Contributions from volume conduction of signals from distant locations can not be excluded, neither in dU nor in NREM.

We recorded LFP from the surface of the parietal cortex (PAC) of mice during NREM sleep in the dark phase and during deactivated state of urethane-anesthesia (dU). Both states were characterized by prominent slow wave activity (Fig. 1A,B). However, inspection of the raw data and a more thorough time–frequency analyses revealed differences in their regularity: in dU, the slow wave activity was prominent, ongoing and clearly visible, while slow waves were more isolated and dispersed during NREM (Fig. 1C). Surface amplitudes of δ tended to be larger in dU (356 ± 67 µV) compared to NREM (204 ± 22 µV; N = 6, t-test, *p* = 0.057). Moreover, the peak power frequencies, averaged from 12 animals, differed between NREM and dU (Fig. 1D). While NREM power peaked exclusively in the δ (1.5–4 Hz) range, dU power peaks were mainly found in the classical slow oscillation (SO) domain (SO, 0.1–1.5 Hz) with δ ranging second. Power spectral densities (PSD) of surface PAC LFP from each of 15 animals revealed similar results: in NREM, power peaks were exclusively within the δ range (peak power at 2.33 ± 0.11 Hz), with no peaks in the SO range. By contrast, in dU, all animals exhibited SO power peaks (0.90 ± 0.04 Hz), and 11 out of 15 also displayed an additional, smaller power peak in the δ range (1.97 ± 0.11 Hz). Amplitudes and neocortical depth profiles also differed between NREM and urethane (Fig. 1E). In dU, large-amplitude SO and δ dominate the upper cortical layers, with a polarity reversal (phase shift) near 0.3 mm below the surface for both frequency ranges, a feature absent in NREM δ waves (Fig. 1F). Interestingly, recordings from the dorsal hippocampus below the parietal cortex revealed that δ waves in NREM have a 180° phase shift across the pyramidal cell layer (pc), which was absent in SO and δ activity during dU (Fig. 1E,F). In summary, both slow oscillation patterns (SO and δ) showed similar amplitude and depth distribution in dU, but markedly differed from δ in NREM regarding regularity, amplitude, and cortical depth profile. This result shows clear differences between drug-free sleep and urethane conditions. Therefore, we conclude that SO/δ in dU and δ in NREM are different types of oscillations.

### Similarities and differences in activated network activity (θ) during natural sleep and under urethane anesthesia

**Fig. 2:**
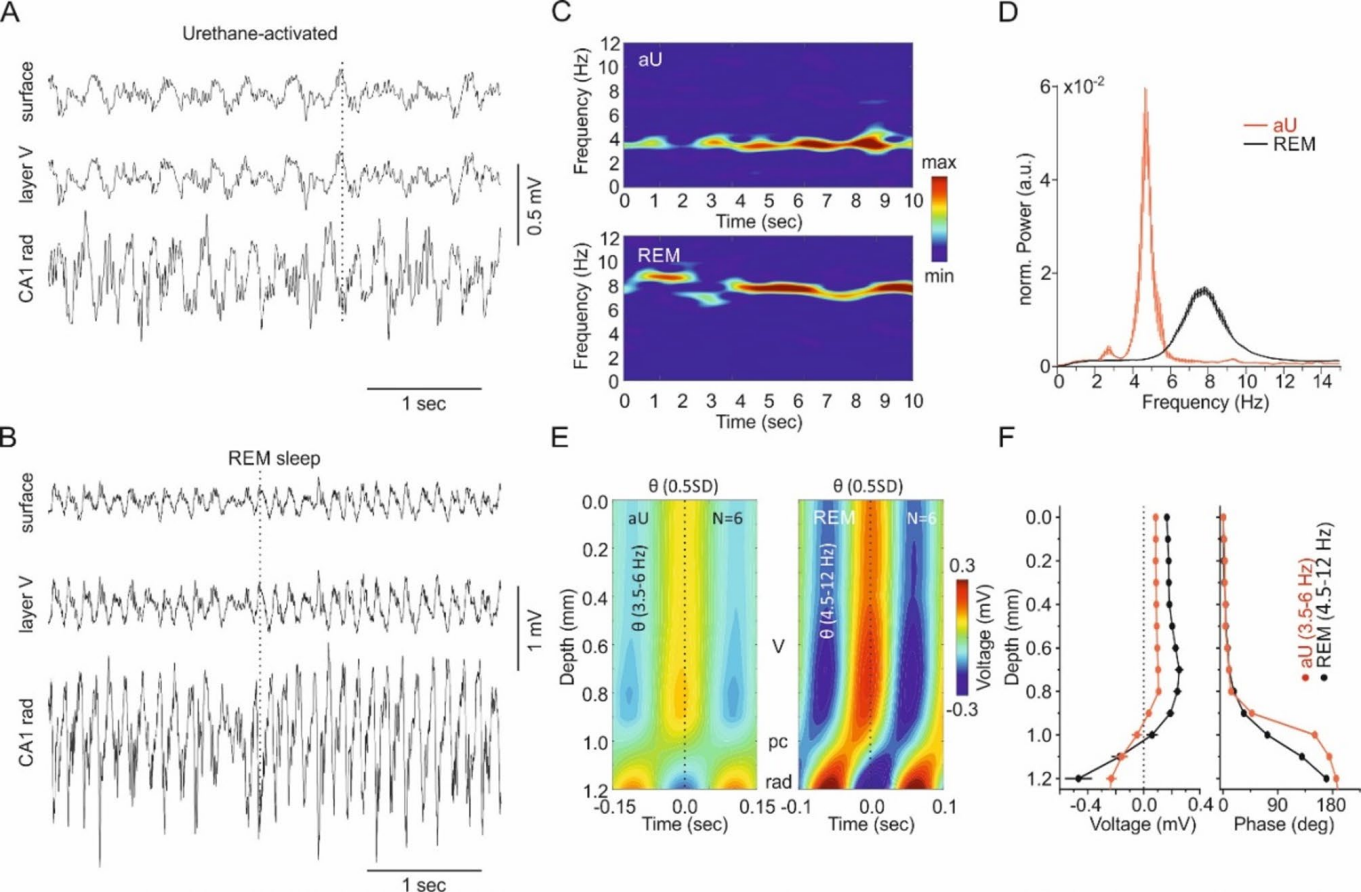
Similarities and differences in activated (θ) network activity during natural sleep and under urethane anesthesia. (**A**) Local field potentials (LFPs) recorded in the activated state of urethane anesthesia (aU) at the surface (surf, upper trace), layer V (middle trace) of parietal cortex (PAC) and stratum radiatum (CA1 rad) of the dorsal hippocampus. (**B**) LFPs recorded in tonic REM sleep at the same depths as in A. Note the different amplitude scales in A and B. (**C**) Time–frequency distribution (TFD) of 10 s periods of CA1 rad LFP during aU (upper graph) and tonic REM sleep (lower graph). (**D**) Mean normalized (norm., see Methods) power spectra at CA1 rad in aU (red, N = 6 animals) and tonic REM sleep (black, N = 10 animals). (**E**) Average voltage depth profiles (N = 6 animals) of 3.5–6 Hz θ in aU (left graph) and 4.5–12 Hz θ in tonic REM sleep (right graph). (**F**) Mean changes in voltage (left, N = 6 animals) and phase (right, N = 6 animals) with depth during aU (red) and REM (black). Note the abrupt phase shift observed in aU compared to the more gradual phase shift in REM sleep.

Both natural REM sleep and aU are characterized by the absence of slow oscillations and presence of a regular θ rhythm. Here we analyzed only tonic REM which dominates natural sleep with 97%^27^. We found that peak θ frequency was lower in aU (4.7 ± 0.1 Hz) than in REM sleep (7.6 ± 0.1 Hz; t-test, *p* < 0.0001, Fig. 2A–D). The amplitude of the θ rhythm at the cortical surface was lower in aU (99 ± 9 µV) compared to REM sleep (203 ± 17 µV, Mann–Whitney test, *p* < 0.0001). In both aU and REM, θ amplitude increased gradually with depth, without any phase shift within the parietal cortex (Fig. 2E,F) in line with the previously reported volume conduction from dorsal hippocampus^26^. This increase appeared less steep in aU compared to REM (Fig. 2F, left). The phase shift across the dorsal hippocampal pyramidal cell layer (pc in Fig. 2E) was abrupt in aU but gradual in REM sleep, as previously described^23,28–32^. In summary, θ oscillations in aU and REM show marked similarities, but also some differences in frequency and in depth-dependent phase and amplitude changes in the dorsal hippocampus.

### Differences in “UP” and “DOWN” periods between dU and NREM

**Fig. 3:**
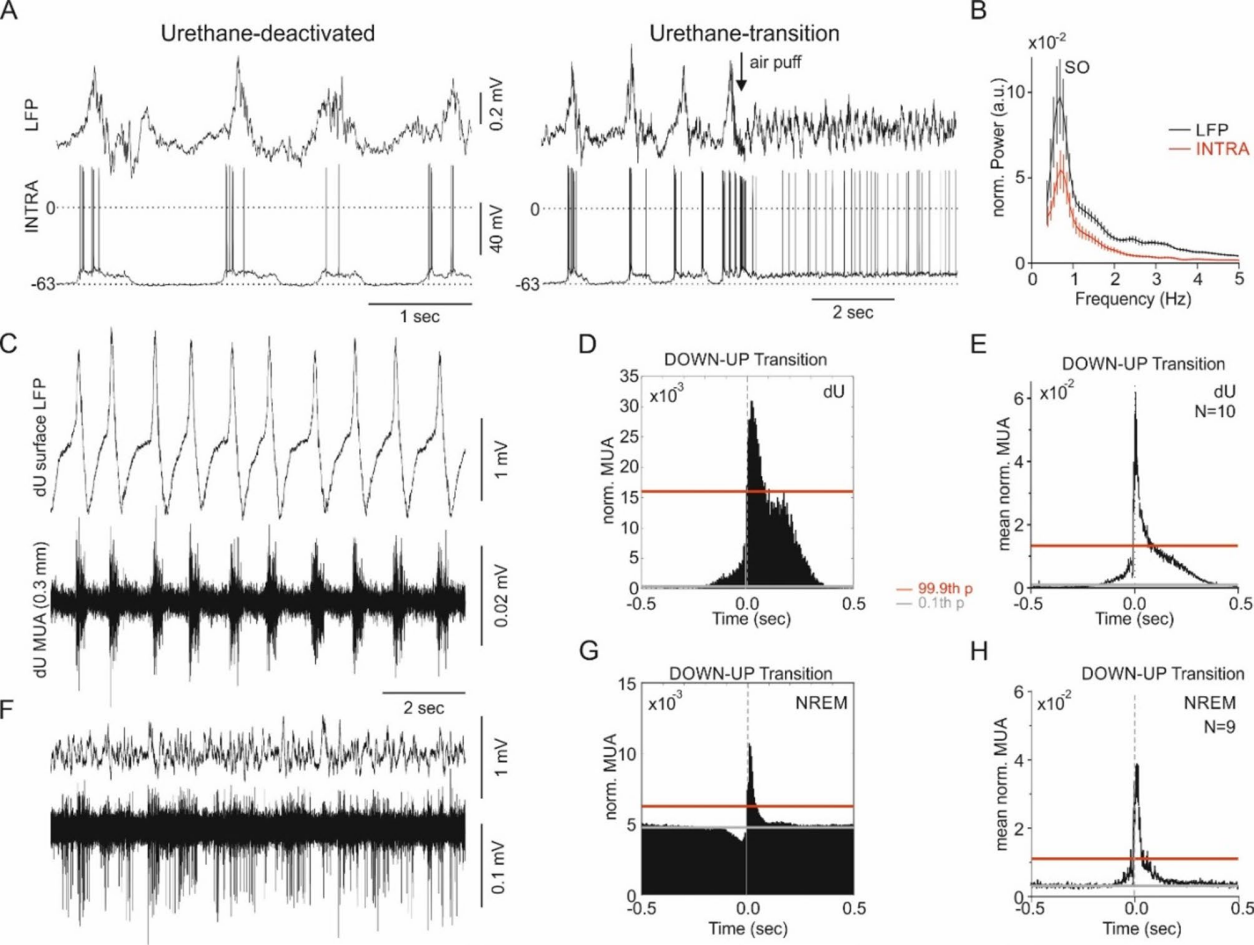
“UP” and “DOWN” periods: differences between urethane anesthesia and NREM sleep*.* (**A**) Local field potential (LFP) and intracellular recording (INTRA) during the deactivated state (dU, left) of urethane anesthesia, and during the transition from dU to the activated state (aU, right) at a cortical depth of 494 µm. Note the depolarization of the membrane potential after the air puff. Absolute values of membrane potential may be more negative than recorded, due to DC potential shifts during the recording period (see Methods). (**B**) Mean normalized (norm., see Methods) power spectral density (PSD) of extracellular LFP (black) and intracellular membrane potential (red) during dU, averaged across 13 animals. (**C**) Raw surface LFP and multi-unit activity (MUA) at 0.3 mm depth during dU. Note the typical bursting activity of MUA in dU, with regular UP and DOWN periods. (**D**) Peri-event histogram (PEH) of normalized MUA (norm., see Methods) centered at the DOWN-UP transition for the example shown in C. (**E**) PEH of mean norm. MUA centered at the DOWN-UP transition, averaged across 10 animals in dU. (**F**) Raw surface LFP and MUA at 0.3 mm depth during NREM sleep. (**G**) PEH centered at the DOWN-UP transition for the example shown in F. Note the differences in background noise and MUA between NREM and dU. (**H**) PEH of mean norm. MUA centered at the DOWN-UP transition, averaged across 9 animals during NREM sleep. Note the smaller amplitude and shorter duration of MUA modulation in NREM compared to dU. For statistics of 0.1th and 99.9th percentiles of the 2 s baseline activity see Methods-Statistics.

Both natural NREM sleep and dU are characterized by periods of increased and decreased cellular activity. This was reflected by fluctuations in the intracellular membrane potential (see Fig. 3A,B for dU), multi-unit activity (MUA), and local field potential (LFP). However, the classical UP and DOWN periods, first described by Steriade’s lab as de- and hyperpolarizing components of SO^21^, were much more pronounced in dU than in NREM, both in intracellular recordings (Fig. 3A) and extracellular MUA (Fig. 3C). In dU, cellular background activity prior to DOWN-UP transitions was close to zero (Fig. 3D,E), whereas it was much higher in NREM, where it was also modulated by these period transitions (Fig. 3G,F).

### Differences in slow oscillations at cortical surface and deep layers between dU and NREM

**Fig. 4:**
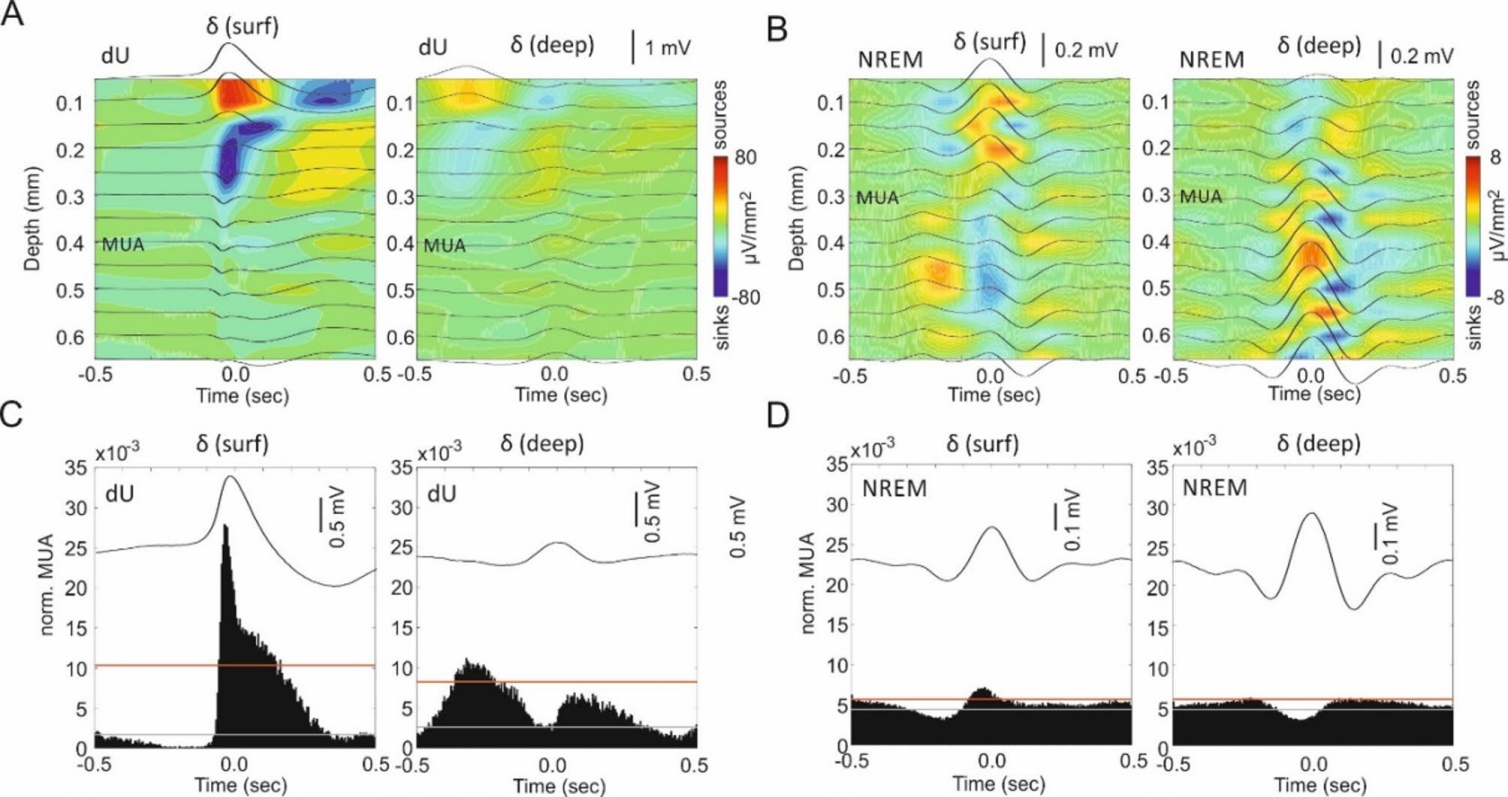
Slow oscillations at cortical surface and deep layers and their unit modulation differ between dU and NREM. (**A**) Current-source density (CSD) of δ (1.5–4.5 Hz) at the surface (surf, left) and deep (right) parietal cortex (PAC) during dU in one representative animal. The superimposed traces show the averaged δ-filtered LFP at the corresponding depth, centered on the δ peaks detected at either the surface (left) or deep PAC (right). Note the large δ amplitude at the surface, the polarity reversal in the upper layers, and the smaller amplitude in deeper layers. Also note the strong sinks and sources in the upper layers, strongest for surface δ peaks and less pronounced for deep δ peaks. (**B**) CSD of surface and deep δ waves in PAC during natural NREM sleep in another animal. Note the five times smaller scale of the superimposed δ-filtered LFPs, and the tenfold weaker sinks and sources, with the strongest located in deeper layers. (**C**) Peri-event histogram (PEH) of normalized (norm., see Methods) multi-unit activity (MUA) at 0.4 mm depth centered on surface δ peaks (left) and deep δ peaks (right) during dU for the same animal as in A. Note the weaker MUA modulation by deep δ waves. (**D**) PEH of norm. MUA at 0.3 mm depth centered on surface δ peaks (left) and deep δ peaks (right) during NREM for the same animal as in B. The corresponding δ waves in C and D are shown. Note the large difference in MUA modulation by δ between dU and NREM, as well as the opposite amplitude relationship of surface and deep δ waves in dU (highest at the surface) and NREM (highest in deeper layers).

Depth distributions of SO and δ activity in the parietal cortex (PAC) were similar in dU (Fig. 1E, F), indicating that they might be generated by a common mechanism. They differed, however, from depth profiles of δ waves in NREM. To investigate the underlying current sources and the modulation of multi-unit activity (MUA), we used dense multi-channel probes with 50 µm electrode spacing. We found an about tenfold higher strength of sinks and sources of δ oscillations in dU compared to NREM, as well as stronger MUA modulation (Fig. 4). Surface δ waves corresponded to increased unit activity in both dU (Fig. 4C, left) and NREM (Fig. 4D, left). In dU, the PEH of unit activity triggered by surface δ waves and by DOWN-UP transitions revealed nearly identical patterns (compare Fig. 4C left with Fig. 3D); however, this was not the case in NREM (compare Fig. 4D left with Fig. 3G). Since previous research reported a suppression of unit discharges during the positive phase of δ waves at the deepest cortical position^33^, we next compared the averaged activity profiles for δ waves recorded at the surface and at 0.7 mm depth. In dU, deep δ waves appeared smaller (Fig. 4A left), with weaker sinks and sources compared to δ waves at the cortical surface (Fig. 4A right). Furthermore, modulation of unit activity was stronger for surface δ waves than for deep δ waves in dU (Fig. 4C). Consistent with previous findings^33^, we found that the positive phase of the deep δ wave coincided with a suppression of unit activity (DOWN) both in dU (Fig. 4C right) and NREM (Fig. 4D right), although this effect was much weaker in NREM. Current source density (CSD) analyses of both surface and deep δ waves in NREM revealed overall weak sinks and sources (Fig. 4B) compared to dU (Fig. 4A), with the strongest sinks/sources occurring in deeper layers, unlike in dU, where the strongest sinks/sources appeared in upper layers (Fig. 4A,B). Please note the ten times scaling difference between Fig. 4A,B. Weak sinks and sources as shown in Fig. 4B for NREM do not rely necessarily on polarity reversal but might by caused by sudden changes in voltage amplitude. Volume conduction can’t be excluded here therefore these findings have to be treated with caution. Combined with the much weaker δ modulation of unit activity in NREM compared to dU (Fig. 4C,D), these findings suggest that δ activity in urethane anesthesia and NREM represent different oscillations.

To compare our results with those of previous reports of recordings from prefrontal regions, we finally examined δ waves in PAC and in the anterior cingulate cortex (ACC) during NREM. In ACC, δ waves were significantly larger than in PAC (NREM: 225 ± 13 µV compared to 143 ± 9 µV, N = 10, t-test, *p* < 0.0001); however, the modulation of unit activity exhibited a similar profile in both regions (not shown). Therefore, we conclude that our findings in NREM are not due to differences between different cortical regions.

## Discussion

In this report, we compare cortical oscillations (SO, δ and θ) in natural sleep and urethane anesthesia. Both states exhibit spontaneous periods with slow waves (during the deactivated state in urethane and in natural NREM sleep), alternating with periods without slow waves but with regular θ activity (during the activated state in urethane and natural REM sleep^10,16^). Our findings highlight similarities and differences between sleep and anesthesia, with notable differences between dU and NREM, and greater similarities between aU and REM.

The main differences between dU and NREM are: (1) regular SO and δ activity in dU versus non-regular, isolated wave-like δ activity in NREM; (2) regular UP (spiking) and DOWN (silence) periods of unit activity in dU, contrasted with weaker unit modulation in NREM, along with the generally higher background activity (incomplete silence during DOWN), sporadic DOWN-UP transitions, and much shorter UP phases; (3) the presence of phase shifts within PAC for SO and δ in dU, versus no phase shift of δ in NREM; and (4) strong current δ sinks and sources in upper PAC layers in dU, contrasted with weak sinks and sources in deeper PAC layers in NREM sleep. The main similarities in θ rhythm between aU and tonic REM sleep are: (1) comparable regularity; and (2) no phase shift in PAC, but phase shifts across the hippocampal pyramidal layer. The main differences are: (1) lower θ frequency in aU compared to tonic REM sleep; (2) lower θ amplitude in aU; and (3) an abrupt hippocampal phase shift in aU compared to a more gradual phase shift in tonic REM sleep. Volume conduction from dorsal hippocampus to the overlaying neocortex has been shown earlier^26^ therefore more detailed conclusions on current generators are to be treated with caution.

The similarities and differences in θ activity between aU and REM sleep have been widely documented in previous studies^10,16,32^. In contrast, differences between dU and NREM have not been extensively characterized. Most studies emphasize the similarity of slow waves in dU and NREM across various animal species and in humans^21,33–40^. However, no prior quantitative frequency analyses of slow waves in dU and NREM in mice have been performed. A previous report in cats found that silent states (in our terminology “DOWN”) were more prominent in ketamine-xylazine anesthesia than during slow wave sleep and their duration was significantly longer in anesthesia compared to natural sleep^41^. Furthermore this study found larger amplitudes of slow oscillations during anesthesia compared to slow wave sleep which is consistent with our present findings in mice^41^.

SO during NREM sleep in rats were reported to be less stable and mainly present during deep slow-wave sleep episodes, in contrast to the more consistent presence of SO in dU^42^. These “deep slow-wave episodes” of NREM sleep, however, were not specifically defined. In our data from mice, we did not observe consistent SO or differences in regularity of δ waves over time during NREM sleep. We can not exclude, however, the possibility that NREM during the dark phase is less deep compared to the light phase.

Another report in rats suggested that SO and δ waves are not separate patterns but rather that δ waves represent the DOWN periods of SO^37^, with cellular DOWN corresponding to slow waves in the deepest cortical layers^33^. During dU, we indeed found similarities between SO and δ, but in NREM there were only δ waves. One possibility is that δ in dU represents a harmonic of the prominent SO, which might account for the similar depth profiles and similar MUA modulation observed for both frequency ranges. Interestingly, however, modulation of MUA by δ was even stronger than modulation by SO in dU.

Due to the absence of SO in natural NREM sleep in our recordings, we compared δ in dU with δ in NREM. In this way, we could test for differences between similar frequency domaines in dU and NREM sleep, rather than comparing the two potentially different oscillations types SO and δ. We note, however, that similar results would be obtained when comparing SO in dU with δ in NREM, given the comparable profile of δ and SO in dU. In line with the absence of SO in NREM, we did not detect unit silence at the cellular level in our experiments in mice during NREM sleep, unlike prior findings in rats (see Fig. 1 in reference 33). Instead, we observed only short UP periods preceded by DOWN periods of lower, yet still significant background activity (Fig. 3G). In dU, both SO and δ strongly modulated cellular activity, whereas δ modulation of unit activity in NREM was much weaker. Notably, we observed a polarity reversal (phase shift) of both SO and δ in PAC during dU but not for δ waves during NREM sleep. A polarity reversal of SO in the rat cortex during dU has been previously reported by Sharma et al. (2010)^43^.

Our findings of striking differences between dU and NREM in PAC may be explained by a recent report from Suzuki and Larkum^5^, who showed that during general anesthesia (including dU), there is: (1) increased input to the upper cortical layers; (2) abolished dendritic transmission to deeper layers; and (3) reduced or abolished thalamic input. In natural NREM sleep, by contrast, dendritic transmission and thalamic inputs are intact, and input to the upper cortical layers is not increased. These differences may account for the generation of distinct slow oscillations between urethane anesthesia and natural sleep.

## Material and methods

The data in this study is described in accordance with ARRIVE guidelines (https://arriveguidelines.org). A total of thirty-four C57/BL6 male mice obtained from Charles River (Sulzfeld, Germany) were used in this study: twelve with chronically implanted electrodes for recordings during sleep, and the remaining twenty-two were used for recordings during urethane anesthesia. Parts of the data recorded during REM sleep and aU have been previously published in a different context^44^.

### Ethics statement

The Governmental Supervisory Panel on Animal Experiments of Baden-Württemberg approved this study (35-9185.81/G-137/17 and 35-9185.81 G-84/13, 35-9185.81/G-44/16). Experiments were conducted in accordance with the U.S. National Institutes of Health’s *Guide for the Care and Use of Laboratory Animals* (1996)^45^ and the European Science Foundation (2001)^46^.

### Animal care

The mice were housed in groups of four, on a 12 h light–dark cycle (with lights on at 8:00 PM), with access to food and water ad libitum. After electrode implantation, animals were housed individually.

### Animal preparation and recordings

#### Chronic electrode implantation

A total of twelve male C57BL/6N mice weighing 24–40 g (12–32 weeks old) were anesthetized with isoflurane in medical oxygen (4% isoflurane for induction, 1.5–2.5% for maintenance, flow rate: 1 L per minute). For analgesia, 0.1 mg/kg of buprenorphine was administered subcutaneously before surgery, four and eight hours post-surgery. Anesthetized animals were mounted on a stereotaxic apparatus (Kopf Instruments) with a custom-made inhalation tube. Body temperature was maintained at 38 °C using a heating pad (ATC-2000, World Precision Instruments). After exposure of the skull, holes of 0.5–1.0 mm in diameter were drilled above the right and left parietal cortex (PAC) and the cerebellum according to stereotaxic coordinates^47^. Two stainless steel watch screws (1 mm diameter, 3 mm length) over the cerebellum served as ground and reference electrode. In nine animals, silicon probes (A1 × 16-3 mm-100–703-CM16LP, NeuroNexus Technologies) were chronically implanted into the right PAC (2.0 posterior to bregma, 1.5 mm lateral); the uppermost electrode was located 100 µm above the cortical surface and the deepest below the CA1 pyramidal cell layer. A different probe type (A1 × 16-3 mm-50-177-CM16LP) was implanted in three additional animals into the right PAC (2.0 posterior bregma, 1.5 mm lateral), with the uppermost electrode located at the cortical surface and the deepest at 750 µm.

#### Electrophysiology in freely moving mice

One week after surgery, recordings began with a 1-h habituation session, followed by several recording sessions of up to 4 h on consecutive days. Room temperature was kept stable at 24 °C. Extracellular signals (LFP) were filtered (1–500 Hz), amplified (RHD2132, Intan Technologies), digitized (2.5 kHz for local field potentials and 20 kHz for multi-unit activity), and stored for offline analysis with custom-written MATLAB (The Mathworks, Inc.) routines. The Intan preamplifier on the animals head contained an in-built 3-D accelerometer to record the animal’s movements synchronously with the LFP.

#### Urethane experiments

Twenty-two male C57BL/6N mice weighing 23–32 g (12–16 weeks old) were initially anesthetized with a mixture of urethane (1.2 g/kg) and ketamine-xylazine (10 mg/kg, 1 mg/kg, i.p.). Solutions were freshly prepared in isotonic (0.9%) NaCl and heated to 38 °C before administration. Ketamine-xylazine dose was ten times lower than usually applied in mouse anesthesia (80–100 mg/kg and 10 mg/kg xylazine) without urethane. In previous experiments we found that the effect of such low doses of ketamine-xylazine without urethane expired within one hour. The initial dose of urethane was also relatively low and its effect did not last for the entire experiment. A higher urethane dose would lead to a higher mortality rate during the experiment and most importantly would not permit suppression of slow waves (deactivated state) and induction of the activated state with θ oscillations by pinching the hind limb^48^. Supplemental doses of urethane (0.2 g/kg, without ketamine-xylazine) were administered throughout the experiment to prevent reflex responses to hind limb pinching (approximately every 1.5 h). The animals were mounted on a stereotaxic frame (Kopf Instruments) and body temperature was maintained at 38 °C. Room temperature was kept at 24 °C. Following skull exposure, holes of 0.5–1.0 mm in diameter were drilled above the right and left PAC according to stereotaxic coordinates^47^. The dura mater was carefully removed and a 125 µm tungsten electrode (MicroProbes) was implanted into the left PAC (2.0 mm posterior to bregma, 1.5 mm lateral, 0.7 mm ventral) for LFP recordings. In some animals, PAC LFPs were recorded ipsilaterally at the right PAC (1.96 mm posterior to bregma, 0.5 mm lateral, 0.75 mm ventral) at a 30° angle to avoid interference with microelectrodes (see below). Extracellular signals from left PAC were filtered (1–500 Hz), amplified (EXT-16DX or EXT 10-2F, npi, Tamm), digitized (20 kHz) using the CED 1401 board, and stored for offline analyses. Recordings started 2 h after the initial application of ketamine-xylazine/urethane mixture, approximately 1 h after the effect of low dose ketamine-xylazine faded out. Intracellular recordings were obtained from right PAC neurons using high-impedance quartz-glass micropipettes (o.d.1.0/i.d.0.5 mm; impedance: 60–120 MΩ) pulled with the P-2000 puller (Sutter Instruments) penetrating the brain under an angle of 15° (2.0 posterior to bregma, 1.5 mm lateral). Recording electrodes were filled with 1 M potassium acetate and slowly lowered in 5 µm steps using the Micropositioner 2660 (Kopf Instruments). Axoclamp 900A (Molecular Devices) in current-clamp mode with a bridge circuit was used to amplify intracellular signals. For unit recordings, signals were filtered (0–10 kHz) and digitized at 20 kHz. DC drifts during intracellular recordings were corrected by comparing the initial potential levels with those after withdrawal of the electrode. We note that the time of DC potential drifts during the recording is difficult to predict, so that absolute potential values may differ from our assigned potentials. Extracellularly recorded multiunit activity (MUA) from the 16-channel probes was obtained by high pass filtering (500 Hz). Spike times were defined by setting a threshold above background noise upon visual inspection of individual MUA signals. Since MUA was obtained from single probe contacts, we did not attempt to identify single unit cell types in these signals.

To induce the θ rhythm characteristics of the activated state (aU) of urethane anesthesia, tail pinches or air puffs^48^ were used to temporarily suppress the slow-wave activity typical for the deactivated state (dU).

### Sleep staging

Sleep–wake stages were manually identified by an experienced senior researcher (JB) based on artifact-free LFP recordings from the surface of PAC during circadian dark phase (light on at 8 pm). PAC was chosen due to the presence of θ oscillations volume conducted from the underlying dorsal hippocampus^26^. θ oscillations are present during locomotion and REM sleep but not in quiet waking, drowsiness or NREM sleep^30,49^. Using a 3D-accelerometer built into the Intan preamplifier board, motion (70%) was separated from immobility (30%). NREM sleep (10%) was characterized by immobility, persistent presence of large amplitude slow waves (δ) or sleep spindles, and absence of θ oscillations. Recordings (4 h each) were performed on up to 7 days. Data from the first recording day was not used due to a low percentage of sleep time in some animals. Occasional slow waves occur also in quiet waking and more often in drowsiness, therefore the onset of NREM sleep was defined by persistent continuous occurrence of large amplitude slow δ waves. REM sleep was defined by immobility and the presence of a continuous θ rhythm following NREM sleep^49,50^ mostly terminated by awakening. To avoid interference with slow-wave sleep, REM sleep onset was marked only after all residual delta wave activity from the preceding NREM sleep episode was absent. REM sleep consists of tonic and phasic sub-stages. Phasic REM (2% of REM sleep time) is characterized by higher θ frequencies^27^. For comparison to urethane θ only tonic REM was used.

### Data analysis

Built-in and custom-written MATLAB (The Mathworks Inc., Natick, MA) routines were used. The power spectrum was calculated using the *pwelch.m* function (Signal Processing Toolbox). Power values were normalized by dividing by the total power (0–200 Hz). Phase time series were extracted using the Hilbert transform (*hilbert.m* function). Filtering into slow oscillation (SO: 0.3–1.5 Hz), δ (1.5–4.5 Hz) and θ (3.5–5 Hz for aU and 5–10 Hz for REM) bands was achieved using the *eegfilt.m* function from the EEGLAB toolbox^51^. The root mean square (RMS) of band-pass filtered EEG was smoothed (100 ms window); the mean and standard deviation (SD) of the smoothed RMS was computed across the total signal length.

#### Depth profiles

The reference signal was taken as the band-filtered LFP from the PAC surface. Peaks of identified cycles of SO, δ or θ (θ: 0.5, SO: 1 or δ: 2 SD above mean RMS) at the PAC surface served as triggers for averaging the filtered signals across the 16 electrode positions of the silicon probe (n > 50 waves). Voltage and phase profiles as a function of recording depth were averaged across animals (Figs. 1E,F and 2E,F).

#### Peri-event spike histograms

Spike time series were computed from 500 Hz high-pass filtered multi-unit activity (MUA). Spike times were identified based on spike amplitudes exceeding a minimum of two times the background noise amplitude. Periods of high spike firing (referred to as “UP”) were detected from the spike time series with a minimum of five spikes per UP and a minimal interval of 10 ms between UP periods. Event times of DOWN-UP transitions corresponded to the first spike time in UP periods. Peri-event histograms (PEH, Fig. 3D,G) summed the number of spikes per bin (5 ms bin size) 0.5 s before and after each DOWN-UP transition. MUA was normalized by dividing the number of spikes per bin by the total number of spikes. The spiking activity from 2 to 1 s prior to the DOWN-UP transition was used to estimate the 99.9 and 0.1 percentiles of baseline activity. Mean PEH, percentiles (Fig. 3E,H), and standard error (SE) were calculated across individual PEHs.

### Histology

After the last experiment, the animals were deeply anesthetized with ketamine-xylazine (30 mg/kg + xylazine 10 mg/kg) and perfused transcardially with PBS followed by 4% paraformaldehyde. The position of the multichannel probes was then verified by staining with DAPI (4,6-Diamidin-2-phenylindol) and fluorescence light microscopy.

### Statistics

Data are expressed as mean ± standard errors of the mean (SEM) unless otherwise stated. The t-test was used for parametric data and the Mann–Whitney test for non-parametric data. A value of *p* < 0.05 was considered statistically significant. Circular data were averaged using the *anglemean.m* function. For further statistics details, see the Data analysis section above. *Peri-event spike histograms*: The spiking activity from 2 to 1 s prior to the DOWN-UP transition was used as baseline activity. Percentiles (99.9 and 0.1) of the baseline activity were defined from these data.

## Data Availability

Data are available from the corresponding authors upon reasonable request.
